# Seasonal Variation, Chemical Composition, and Analgesic and Antimicrobial Activities of the Essential Oil from Leaves of *Tetradenia riparia* (Hochst.) Codd in Southern Brazil

**DOI:** 10.3390/molecules15085509

**Published:** 2010-08-10

**Authors:** Zilda Cristiani Gazim, Ana Carolina L. Amorim, Ana Maria C. Hovell, Claudia Moraes Rezende, Izalina Ansilieiro Nascimento, Gilberto Alves Ferreira, Diógenes Aparício Garcia Cortez

**Affiliations:** 1 Programa de Pós-graduação em Ciências Farmacêuticas, Universidade Estadual de Maringá, Maringá, Paraná, Brazil; 2 Instituto de Química, Universidade Federal do Rio de Janeiro, Centro de Tecnologia; 3 Universidade Paranaense, Unipar, Umuarama, Paraná, Brazil; 4 Departamento de Farmácia, Universidade Estadual de Maringá, Av. Colombo, 5790, 87020-900, Maringá, Brazil

**Keywords:** *Tetradenia riparia* (Hochst.) Codd, seasonal variation, GC-MS, analgesic activity, antimicrobial activities

## Abstract

The seasonal variation of the chemical composition of the essential oil from fresh leaves of *Tetradenia riparia* (Hochst.) Codd grown in southern Brazil was analyzed by GC-MS, and the analgesic and antimicrobial activities of this oil were assayed. The yield of essential oil ranged from 0.17% to 0.26%, with the maximum amount in winter and the minimum in spring. The results obtained from principal components analysis (PCA) revealed the existence of high chemical variability in the different seasons. The samples were clearly discriminated into three groups: winter, autumn, and spring-summer. Samples collected during winter contained the highest percentages of calyculone (24.70%), abietadiene (13.54%), and viridiflorol (4.20%). In autumn, the major constituents were ledol (8.74%) and *cis-*muurolol-5-en-4-α-ol (13.78%). Samples collected in spring-summer contained the highest percentages of fenchone (12.67%), 14-hydroxy-9-epi-caryophyllene (24.36%), and α-cadinol (8.33%). Oxygenated sesquiterpenes were predominant in all the samples analyzed. The observed chemovariation might be environmentally determined by a seasonal influence. The essential oil, when given orally at a dose of 200 mg/kg, exhibited good analgesic activity on acetic acid-induced writhing in mice, inhibiting the constrictions by 38.94% to 46.13%, and this effect was not affected by seasonal variation. The antimicrobial activity of the essential oil against the bacterial strains: *Staphylococcus aureus, Bacillus subtilis*, *Enterococcus faecalis*, *Escherichia coli*, *Salmonella enterica*, *Pseudomonas aeruginosa*, *Klebsiella pneumonia*, *Proteus mirabilis, Morganella morganii,* and *Enterobacter cloacae,* and the pathogenic fungus *Candida albicans* was assessed by the disc diffusion method and determination of the minimum inhibitory concentration. The results obtained, followed by measurement of the minimum inhibitory concentration (MIC), indicated that *S. aureus*, *B. subtilis,* and *Candida albicans* were the most sensitive microorganisms, showing largest inhibition, and the lowest MIC values varied from 15.6 to 31.2 µg/mL, 7.8 to 15.6 µg/mL, and 31.2 to 62.5 µg/mL, respectively.

## 1. Introduction

*Tetradenia riparia* (Hochst.) Codd [Lamiaceae] is an herbaceous shrub that occurs throughout tropical Africa [1]. *T. riparia* possesses a variety of medicinal properties. In South Africa, *T. riparia* has traditionally been used in the treatment of cough, dropsy, diarrhea, fever, headaches, malaria, and toothache [2]. In Brazil, *T. riparia* was introduced as an exotic ornamental plant and is grown in parks, home gardens, and orchards in the state of São Paulo. In Brazil it is popularly known as incenso, lavândula, limonete, pluma-de-névoa, or falsa mirra, and is mainly used as an ornamental [3]. 

The essential oil from the leaves and stems of *T. riparia* from South Africa was analyzed by GC/MS, and 35 components were identified. The main constituents were α-terpineol (22.6%), fenchone (13.6%), β-fenchyl alcohol (10.7%), β-caryophyllene (7.9%), and perillyl alcohol (6.0%) [2]. Essential oil from *T. riparia* plants grown in Kenya showed *sensu stricto* repellent activity against *Anopheles gambiae* [4] and moderate anti-malarial activity against two strains of *Plasmodium falciparum* [2]. Essential oil and diterpenes isolated from *T. riparia* leaves showed antibacterial and antifungal activity [5]. The essential oil of *T. riparia* showed insecticidal properties against Zabrotes subfasciatus (Col., Bruchidae) infesting dried pinto beans (Fabales, Leguminosae) [6]. A diiterpene diol isolated from a chloroform extract of leaves of *T. riparia* exhibited significant antimicrobial activity against several bacteria and fungi; the minimum inhibitory concentration (MIC) for those microorganisms which were inhibited ranged from 6.25 to 100 μg/mL [7]. This isolated diterpene diol also showed antispasmodic activity [8].

The anti-inflammatory and antimicrobial activities of essential oils have formed the basis of many applications in pharmaceuticals, alternative medicine, and natural therapies [9]. Efforts have also been made to explore the potential of some essential oils for the treatment of infectious diseases, in order to replace standard pharmaceutical remedies [10]. The quality of essential oil depends on different factors. Among them are the chemotype and biotype of the plant, as well as the climatic conditions. There are no existing data concerning the seasonal variation of any of the constituents of *T. riparia.* A study of the influence of different periods of ripening on the chemical composition of essential oil from fresh leaves of this species is therefore useful [11]. 

We have investigated and now report the chemical composition of the essential oil isolated from fresh leaves of *T. riparia* cultivated in southern Brazil, as affected by different growing seasons, together with their anti-inflammatory and antimicrobial activities.

## 2. Results and Discussion

### 2.1. Oil yield (%)

**Figure 1:**
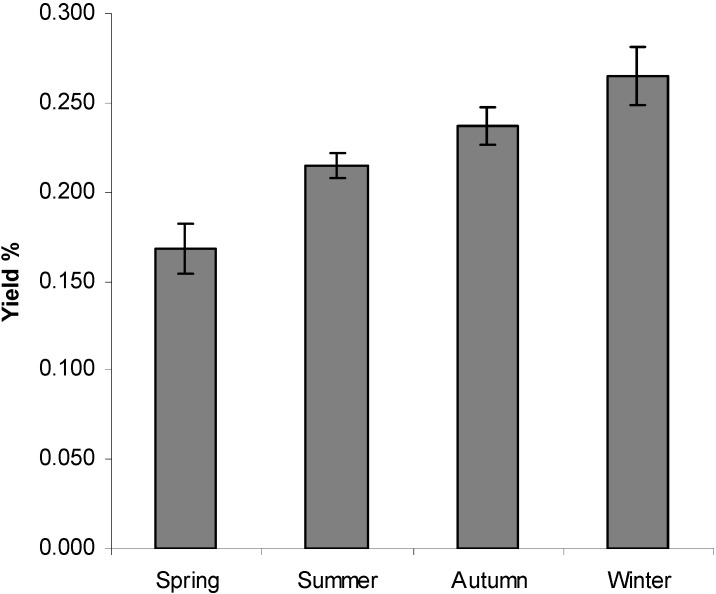
Yield of essential oils from fresh leaves of *Tetradenia riparia* collected monthly from September 2006 through August 2007, using Dunn's multiple comparison test (P = 0.0006).

**Figure 2:**
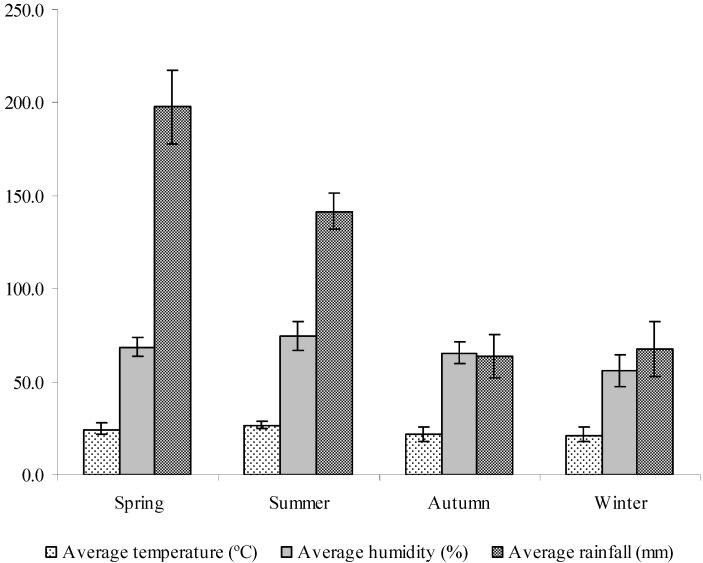
Means and standard deviations of the temperature (°C), rainfall (mm), and relative humidity (%) by season, from September 2006 through August 2007.

As shown in Figure 1, the yield of oil was affected by seasonal changes. The content of the essential oils was also distributed unevenly among the seasons. The largest amount of oil in *T. riparia* was found during winter (0.265% ± 0.0), and the oil content decreased significantly (p < 0.05) in spring to 0.168% ± 0.02. During the spring, the rainfall was much higher than in the other seasons. Temperature and humidity did not change significantly during the study period (Figure 2). Studies conducted by Lima *et al*. [26] showed that the amount of special metabolites produced during the development of the plant can be affected by radiation (high or low), temperature (high or low), precipitation (high, low, and total dry matter), winds, altitude, soil, and time of harvest, among other factors. Temperature, relative humidity, the total duration of exposure to sun, and wind patterns have a direct influence, especially in species that have histological structures for the storage of essential oil on the leaf surface [27]. Hussain *et al*. [28] observed that temperature influenced the decrease in oil yield of *O. basilicum,* with the highest yields in the winter (0.8%) and a decrease in summer to 0.5%.

### 2.2. Identification and quantification of T. riparia essential oil

In total, 36 compounds were identified, accounting for 95.0–99.6% of the volatile constituents (Table 1) in the leaves. Oxygenated sesquiterpenes were the dominant compounds, comprising 45.18–64.33% of the oil, and they consisted mainly of 14-hydroxy-9-epi-caryophyllene (18.27–24.36%), *cis*-muurolol-5-en-4-α-ol (7.06–13.78%), α-cadinol (5.36–8.33%), and ledol (4.39–8.74%). The percentage of oxygenated diterpenes was 12.11–25.73%, represented by calyculone (11.57–24.70%); and the main diterpene hydrocarbon constituent was abietadiene (5.51–13.54 %). Oxygenated monoterpenes comprised 5.37–19.52% of the total essential oil, and the major constituent was fenchone (2.40–12.67%). The essential oil of *T. riparia* cultivated in the Kirstenbosch National Botanical Garden in South Africa differs in composition from the oil grown in southern Brazil. As a group, the monoterpenes represented 69.0% of the oil. Major components were α-terpineol (22.6%), fenchone (13.6%), fenchyl alcohol (10.7%), β-caryophyllene (7.9%) and perillyl alcohol (6.0%). Sesquiterpene hydrocarbons and alcohols accounted for 29.1% of the composition [2]. It was reported in [4] that oil of *T. riparia* grown in Kenya had monoterpenes as major constituents. Among these, a fenchone (64.82%) among the oxygenated, and limonene (2.02%) and 1,8-cineole (1.5%) were prominent among the hydrocarbons. The observed differences in the constituents of essential oils of *T. riparia* grown in Africa may be due to different environmental and genetic factors, different chemotypes, and the nutritional status of the plants.

**Table 1:** Chemical composition of *Tetradenia riparia* essential oil.

Peak	^A^Compounds			% Composition			Methods ofIdentification
		RI^a^	Spring	Summer	Autumn	Winter	
	*Monoterpene Hydrocarbons*						
1	Limonene	1047	0.90 ± 0.13^c^	3.69 ± 1.06^a^	t	2.32 ± 0.80^b^	i.j.
	*Oxygenated Monoterpenes*						
2	Fenchone	1051	2.40 ± 0.18^b^	12.67 ± 1.04^a^	3.42 ± 0.46^b^	5.65 ± 0.58^b^	i.j.
3	*endo*-Fenchol	1093	0.77 ± 0.17^b^	1.90 ± 0.65^a^	0.87 ± 0.10^b^	1.11 ± 0.55^b^	i.j.
4	Camphor	1108	0.90 ± 0.20^bc^	2.68 ± 0.76^a^	1.15 ± 0.19^bc^	1.67 ± 0.79^b^	i.j.
5	Borneol	1119	0.73 ± 0.09^b^	1.43 ± 0.32^a^	0.90 ± 0.19^b^	1.09 ± 0.48^b^	i.j.
6	α-Terpineol	1131	0.57± 0.09^a^	0.83 ± 0.17^a^	0.73 ± 0.20^a^	0.72 ± 0.24^a^	i.j.
	*Sesquiterpene Hydrocarbons*						
7	δ-Elemene	1360	t	0.12 ± 0.02^a^	0.15 ± 0.02^a^	t	i.j.
8	α-Copaene	1377	0.53 ± 0.27^c^	1.17 ± 0.26^a^	1.32 ± 0.31^a^	t	i.j.
9	β-Elemene	1395	0.20 ± 0.03^a^	0.27 ± 0.08^a^	0.40 ± 0.08^a^	0.33 ± 0.00^a^	i.j.
10	α-Gurjunene	1400	0.25 ± 0.06^b^	0.46 ± 0.12^b^	0.73 ± 0.16^a^	t	i.j.
11	β-Caryophyllene	1425	0.38 ± 0.06^bc^	0.63 ± 0.20^b^	1.26 ± 0.26^a^	0.34 ± 0.07^bc^	i.j.
12	α *-trans*-Bergamotene	1436	1.57± 0.20^c^	3.33 ± 0.09^b^	4.78 ± 0.65^a^	1.08 ± 0.10^d^	i.j.
13	*allo*-Aromadendrene	1445	0.28± 0.06^bc^	0.34 ± 0.08^bc^	0.90 ± 0.19^a^	0.45 ± 0.11^b^	i.j.
14	Bicyclogermacrene	1495	0.51± 0.09^a^	0.55 ± 0.10^a^	0.80 ± 0.16^a^	0.46 ± 0.28^a^	i.j.
15	α-( *E,E*)-Farnesene	1504	0.22 ± 0.04^b^	0.29 ± 0.08^b^	0.50 ± 0.09^a^	t	i.j.
16	γ-cadinene	1511	0.2 ± 0.03^a^	0.18 ± 0.04^a^	0.21 ± 0.03^a^	t	i.j.
17	δ-Cadinene	1528	0.85 ± 0.22^c^	1.48 ± 0.33^b^	1.76 ± 0.27^a^	0.32 ± 0.05^d^	i.j.
	*Oxygenated Sesquiterpenes*						
18	*cis*-Muurolol-5-en-4-α-ol	1535	13.2 ± 0.18^a^	11.74 ± 0.09^a^	13.78 ± 0.56^a^	7.06 ± 0.19^b^	i.j.
19	Ledol	1541	7.11± 0.22^a^	7.00 ± 0.24^a^	8.74 ± 0.84^a^	4.39 ± 2.59^b^	i.j.
20	Caryophyllenyl alcohol	1544	0.53± 0.19^a^	0.39 ± 0.02^a^	0.47 ± 0.06^a^	0.37 ± 0.06^a^	i.j.
21	Spathulenol	1576	0.16 ± 0.02^b^	0.10 ± 0.02^b^	0.15 ± 0.03^b^	0.33 ± 0.11^a^	i.j.
22	Globulol	1589	2.81± 0.96^a^	3.16 ± 0.70^a^	3.97 ± 1.31^a^	1.16 ± 0.32^b^	i.j.
23	Viridiflorol	1592	0.93 ± 0.26^b^	0.50 ± 0.20^b^	1.11 ± 0.18^b^	4.20 ± 0.99^a^	i.j.
24	Guaiol	1599	1.54 ± 0.18^b^	1.24 ± 0.19^b^	1.83 ± 0.41^b^	3.27 ± 0.54^a^	i.j
25	*epi-α*-Muurolol	1656	0.41± 0.10^a^	0.27 ± 0.07^a^	0.22 ± 0.06^a^	0.36 ± 0.12^a^	i.j
26	α-Cadinol	1669	8.33 ± 1.25^a^	5.36 ± 0.84^b^	6.24 ± 1.35^b^	7.11 ± 1.54^ a^	i.j.
27	14-Hydroxy-9- *epi*-caryophyllene	1688	24.36 ± 2.68^a^	18.27 ± 0.19^ab^	20.34 ± 2.59^ab^	t	i.j.
28	(2 *Z*,6*E*)-Farnesol	1709	1.67 ± 0.41^a^	1.16 ± 0.28^ab^	1.28 ± 0.25^ab^	0.73 ± 0.23^c^	i.j.
29	Guaiol acetate	1716	0.69 ± 0.06^b^	0.41 ± 0.03^b^	0.53 ± 0.11^b^	1.82 ± 0.73^a^	i.j.
30	*iso*-Longifolol	1728	0.28 ± 0.11^b^	0.14 ± 0.03^b^	0.21 ± 0.04^b^	1.50 ± 0.32^a^	i.j.
31	Oplopanone	1753	0.16 ±0.01^b^	0.15 ± 0.02^b^	0.21 ± 0.06^a^	t	i.j.
32	14-Hydroxy- *α*-muurolene	1782	1.22 ± 0.01^b^	0.22 ± 0.14^c^	0.73 ± 0.13^c^	7.44 ± 2.17^a^	i.j.
33	8-Cedren-13-ol acetate	1799	t	t	t	0.80 ± 0.32^a^	i.j.
34	n.i	1812	0.28 ± 0.03^b^	t	t	0.84 ± 0.36^a^	i.j.
35	n.i	1831	0.65 ± 0.36^b^	0.17 ± 0.01^bc^	0.39 ± 0.00^bc^	3.80 ± 1.58^a^	i.j.
	*Diterpene Hydrocarbons*						
36	Abietadiene	2017	6.85 ± 0.66^b^	5.51 ± 1.50^b^	6.33 ± 1.07^b^	13.54 ± 2.18^a^	i.j.
	*Oxygenated Diterpenes*						
37	Manoyl oxide	2096	1.61 ± 1.59a	0.41 ± 0.09b	0.81 ± 0.25a	0.70 ± 0.23b	i.j.
38	n.i	2141	0.31 ± 0.08^a^	0.21 ± 0.03^b^	0.20 ± 0.05^b^	0.34 ± 0.06^a^	i.j.
39	Calyculone	2217	15.64 ± 1.11^b^	11.57 ± 0.38^b^	12.58 ± 1.67^b^	24.70 ± 1.34^a^	i.j.
	**Total identified**		98.76	99.62	99.41	95.02	

Values are mean ± standard error of CG area (%) the essential oil of *T. riparia* obtained in the seasons. Values in the same line with Different subscript are significantly different within seasons; t: (p < 0.05); i = Identification based on retention index; j = identification based on comparison of mass spectra; n.i: unidentifiel compound; All data represent the mean values of two independent duplicates and statistical analysis were performed by analysis of variance (ANOVA) using; BIOESTAT 5.0 (Stat Soft Inc., Tulsa, OK, USA) software. A probability value at p < 0.05 was considered statistically significant. Data are presented; as mean values ± standard deviation calculated; ^A^Compound listed in order of elution from a DB-5 column.

### 2.3. Seasonal variability

The data from the present analysis demonstrated that the content of the essential oil varied significantly (p < 0.05) with season (Table 1). Oxygenated sesquiterpenes that varied mostly with season were 14**-**hydroxy**-**9**-***epi***-**caryophyllene (maximum 24.36% in spring, and absent in winter), and *cis*-muurolol-5-en-4-α-ol with a maximum of 13.78% in autumn and a minimum of 7.06% in winter. Of the oxygenated diterpenes, the content of calyculone was highest during winter (24.70%) and lowest in summer (11.57%). For abietadiene, the maximum was 13.54% in winter and the minimum 5.51% in summer. Of the oxygenated monoterpenes, fenchone was the major constituent, with a maximum of 12.67% in summer and a minimum of 2.40% in spring. The percentages of these main constituents changed irregularly during the seasons. These changes have a direct impact on the quantity and composition of the oil. The changes in composition reflect the significant changes that occurred from September 2006 through August 2007, of *T. riparia* acclimated in southern Brazil. This study is the first to examine the influence of seasonal variation on the chemical composition of *T. riparia* essential oils. 

These results are in good agreement with [28], who reported variations in the composition of essential oil obtained from *Ocimum basilicum* at different seasons of the year. The contents of oxygenated monoterpenes and oxygenated sesquiterpenes in the essential oils were highest during winter (80.9%) and lowest during summer (74.3%). Inversely, sesquiterpene hydrocarbons reached a maximum of (24.3%) in summer and a minimum in winter (16.0%). In another experiment [29] on the change in chemical composition depending on the seasonality of the essential oil of *Myrcia salzmannii* Berg. (Myrtaceae), α-pinene was present in the highest concentrations from April to December, and was absent in February. β-Caryophyllene was always present in higher concentrations, showing the maximum concentration in February 2001 and the minimum in April 2003. Another prominent component in the oil was α-humulene; except for February 2001 and April 2003, its concentration was relatively high, between 10.3% and 13.2%.

### 2.4. Antimicrobial activity

The antimicrobial activity of the essential oils obtained from *T. riparia* collected seasonally was assayed against nine pathogenic microorganisms (Table 2). The results obtained from disc diffusion method, followed by measurement of the minimum inhibitory concentration (MIC), indicated that *S. aureus*, *B. subtilis,* and *Candida albicans* were the most sensitive microorganisms. They showed the largest inhibition zones, from 7.0 to 22.3 mm, 8.3 to 19.3 mm, and 19.3 to 22.3 mm respectively. They also showed the lowest MIC values, from 15.6 to 31.2 µg/mL, 7.8 to 15.6 µg/mL, and 31.2 to 62.4 µg /mL, respectively. This activity can be attributed to the presence of monoterpenes, sesquiterpenes, and diterpenes, as previously reported by [30,31,32]. The activity against *S. aureus, B. subtilis*, and *C. albicans* was highest in summer. 

**Table 2:** Season variation in antimicrobial activity of *Tetradenia riparia* essential oil.

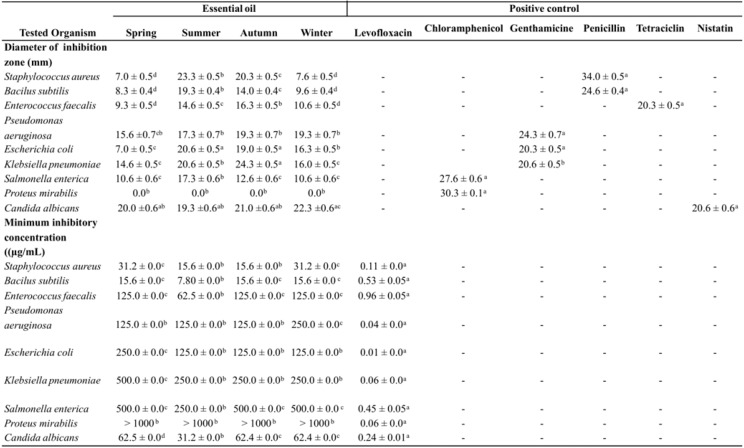

Values are means ± standard deviation for triplicate experiments and statistical analysis of the data were performed by analysis of variance (ANOVA) using BIOESTAT 5.0 (Stat Soft Inc., Tulsa, OK, USA) software. Values in the same line with different subscript are significantly different (p < 0.05).

The essential oil of *Lavandula pedunculata* shows activity against different strains of fungi involved in candidosis, dematophytosis, and aspergillosis. The oils contain a high percentage of oxygenated monoterpenes, the main compounds being 1,8-cineole (2.4–55.5%) and fenchone (1.3–59.7%) [33]. Other lavender oils inhibited the growth of both methicillin-sensitive and methicillin-resistant *Staphylococcus aureus* (MSSA and MRSA) [34]. The results of the bioassays showed that essential oils exhibited good antibacterial activity against all Gram-positive bacteria. According to several authors, Gram-negative bacteria appeared to be less sensitive to the action of many other plant essential oils. This higher resistance among Gram-negative bacteria could be due to the highly hydrophilic cell membrane of this bacterial group; the cell membrane of Gram-positive bacteria may facilitate penetration by hydrophobic compounds [35]. However, further investigation to establish how components interact to provide the antimicrobial activity is needed.

### 2.5. Analgesic activity

**Figure 3:**
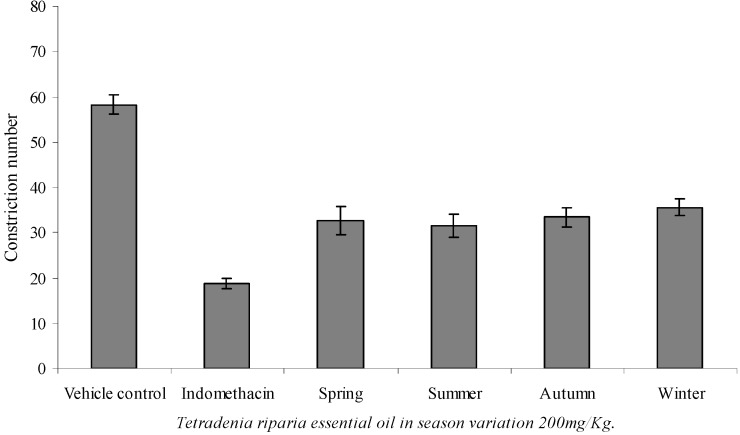
Effect of the essential oil of *Tetradenia riparia* and indomethacin (10 mg/kg) on mouse abdominal constrictions induced by acetic acid (0.6%, i.p.). 200 mg/kg p.o. Data are expressed as mean ± SEM (n = 8-10 animals per group). P < 0.05 (Student’s t test) as compared to the vehicle control group.

*Tetradenia riparia* is used in popular medicine as a remedy against a wide range of diseases, including malaria, angina, yaws, helminth-induced diseases, gastroenteritis, gonorrhea, diarrhea, dental abscesses, headache, and several kinds of fevers and aches [7]. The antinociceptive activity of the essential oil from the leaves of this plant has never been measured. The antinociceptive activity as assessed by the mouse constriction test is illustrated in Figure 3. The essential oil, given orally at a dose of 200 mg/kg, inhibited constrictions by 38.94 to 46.13%, and this activity was not affected by seasonal variation. The majority compound of the essential oil from *T. riparia* is 14-hydroxy-9-*epi*-caryophyllene. Caryophyllene derivatives and essential oils containing compounds of these classes showed significant analgesic and anti-inflammatory activity [36,37]. The essential oil of *T. riparia* is a potential natural product for developing a phytomedicine with analgesic properties.

### 2.6. Principal components analysis (PCA)

**Figure 4:**
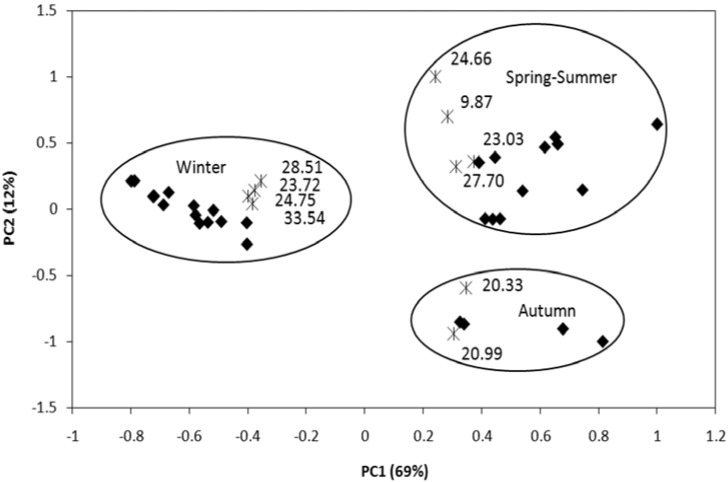
Biplot of PCA scores and loadings for the GC-FID volatile profile of the essential oil from leaves of *Tetradenia riparia* (Hochst.) samples (♦), using 10 selected variables (*).

The data obtained through the automatic alignment of the areas versus retention time peak of 34 chromatograms were evaluated by PCA. A PLS-DA model was constructed to identify the most important variables for discrimination among samples from different seasons. Three retention time showed regression coefficient higher in PLS-DA were selected for each of the four seasons, giving a total of 10 retention times. Using a matrix of 34 small (samples) by 10 (retention times selected) was obtained ACP shown in Figure 4.

The ten variables with the highest absolute regression coefficients are listed in Table 3. A PCA model was constructed with these ten variables, and the biplot of scores and loadings is shown in Figure 4. The samples were clearly discriminated into three groups: winter, autumn, and spring-summer. Spring and summer samples were not discriminated from each other. In PC1, representing 69% of total data variability, the winter samples were separated from the others; while in PC2, representing 12% of total data variability, the autumn samples were separated from the other two classes. The variables which were responsible for the separation of samples collected in the same season or group of seasons are shown inside their respective circles. The best view of the separation obtained was obtained by comparison with the evaluation of peak area and the TR of essential oil components of *T. riparia* in different seasons (Figure 4). It was evident that the separation occurred due to the larger amounts of these compounds that were separated. For example, *cis*-muurolol-5-en-4-α-ol (13.78%) and ledol (8.74%) showed the highest concentrations in autumn. Viridiflorol (4.20%), abietadiene (13.54%), and calyculone (24.70%) showed the highest concentrations in winter. L-fenchone (12.67%), α-cadinol (8.33%), and 14**-**hydroxy**-**9**-***epi***-**caryophyllene (24.36%) also showed the highest concentrations in spring-summer (Table 3). 

**Table 3:** Retention times (RT) for the substances selected as most important for the discrimination of the essential oil of *Tetradenia riparia* (Hochst.), collected during the seasonal variation.

Season	RT (min)	Chemical Identification
Spring-Summer	9.87	L-fenchone
	23.03	Caryophyllenyl alcohol
	24.66	α-Cadinol
	27.70	14-Hydroxy-9- *epi*-caryophyllene
Autunm	20.33	*cis*-Muurolol-5-en-4-α-ol
	20.99	Ledol
Winter	23.72	n.i
	24.75	Viridiflorol
	28.51	Abietadiene
	33.54	Calyculene

Multivariate analysis is used to indicate which compounds are most important in generating the observed groups. These compounds may be present in small or large quantities in the samples; a compound that is present in small quantity may well be the most important in discriminating between *T. riparia* collected in different seasons. This is the main reason for using multivariate analysis, to discover minor components which are important for discrimination. This was the case for caryophyllenyl alcohol (0.53%), found in spring-summer. 

PCA allows the graphical visualization of the entire data set, even when the number of samples and variables is high. These algorithms are helpful primarily in increasing understanding of the data set, examining the presence or absence of natural groupings among the samples [38,39,40,41]. 

## 3. Experimental

### 3.1. Plant materials

*Tetradenia riparia* samples were collected monthly between September 2006 and August 2007 in Umuarama (23º 46.225’S 53º16.730’W, 391 m), state of Paraná, Brazil. The plant was identified by Professor Ezilda Jacomasi of the Department of Pharmacy of Paranaense Univeresity (UNIPAR), Paraná. A voucher specimen is deposited at the UNIPAR Herbarium (code number 2502). The essential oil was extracted from the fresh leaves. The mean values for maximum and minimum temperature (°C), rainfall, and relative humidity by season between September 2006 and August 2007 are described in Figure 1.

### 3.2. Chemicals and reagents

All solvents used were of analytical grade. Homologous series of C7-C25 *n*-alkane and *n*-nonadecane reference chemicals used for identification were obtained from Sigma-Aldrich Chemical Co. (St. Louis, MO, USA). All other chemicals, all of analytical grade, *i.e.* anhydrous sodium sulfate, n-pentane, dichloromethane, methanol, and dimethyl sulfoxide (DMSO) used in this study were purchased from Merck (Darmstadt, Germany), unless stated otherwise. All culture media and standard antibiotic discs of gentamicin (10 µg); chloramphenicol (30 mg), tetracycline (30 mg), and penicillin G (10 U) were purchased from LaborClin Products for Laboratories Ltda. Nystatin (100 UI) was obtained from the control center and diagnostic product Ltda, and levofloxacin from Sigma-Aldrich.

### 3.3. Preparation of the samples

Essential oils were obtained from fresh leaves (60 g) by steam distillation in a Clevenger apparatus [2,6] for 3 h with 600 mL of water. At the end of each distillation, the oils were collected and dried with anhydrous Na_2_SO_4_, transferred to glass, and stored in a freezer during the period in which the experiment. The distillations were performed in triplicate.

### 3.4. GC-MS and GC-FID analysis

The GC-MS analyses were performed using an Agilent 5973N GC-MS System operating in EI mode, equipped with a DB-5 capillary column (30 m × 0.25 mm × 0.25 µm, Agilent, Palo Alto, CA, USA) was used to inject 1 µL of a solution sample. The initial temperature of the column was 80 °C. The column was heated gradually to 260 °C with a 4 °C/min rate. The injector (splitless, 0.5 min), and transfer line temperature were held at 260 and 280 °C, respectively. He (1.0 mL/min) was used as the carrier gas. Together with the sample, *n*-nonadecane was added as an internal standard. The same temperature program was used for GC-FID. The identification of the compounds was based on comparison of their retention indices (RI) [13] obtained using various *n*-alkanes (C7 - C25). Also, their EI-mass spectra were compared with the Wiley Library spectra and the literature [14,15]. 

### 3.5. Analgesic activity

#### 3.5.1. Animals and drugs

We used albino Swiss mice (20–25 g) of both sexes from the LASS Bio breeding unit (Faculty of Pharmacy, UFRJ). All animals were kept in standardized conditions, and were given only water *ad libitum* for 12 h before the experiment. The animal experiments were performed according to the ‘‘Principles of Laboratory Animal Care and Use in Research’’ (Colégio Brasileiro de Experimentação Animal-COBEA/Instituto de Biofísica Carlos Chagas Filho- IBCCF^O^, Brazil), based on international guidelines for the care and use of laboratory animals and ethical guidelines for investigation of experimental pain in conscious animals [17]. The essential oil was administered orally (0.1 mL/20 g) as a suspension in Tween 80-ethanol-H_2_O (1:1:10) (vehicle). The essential oil was administered at a dose of 200 mg/kg, and indomethacin (10 mg/kg) was used as the positive control. 

#### 3.5.2. Acetic acid-induced abdominal constriction in mice

The peripheral antinociceptive activity was determined *in vivo* using the mouse abdominal constriction test induced by 0.6% acetic acid (0.1 mL/10 g; i.p.) [18]. The essential oil was administered 1 h before the noxious stimulus. Ten minutes after i.p. injection of the acetic acid, the number of constrictions per animal was recorded for 20 min. Control animals received an equal volume of the vehicle. 

### 3.6. Antimicrobial activity

#### 3.6.1. Microorganisms used and growth conditions

The antimicrobial activity of the essential oil from *T. riparia* leaves was evaluated using the fungus *Candida albicans* (ATCC 10231) and the bacteria *Staphylococcus aureus* ATCC 6538, *Bacillus subtilis* ATCC 6623, *Escherichia coli* ATCC 8739, *Pseudomonas aeruginosa* ATCC 9027, *Enterococcus faecalis* ATCC 29212, *Proteus mirabilis* ATCC 25933, *Klebsiella pneumoniae* ATCC 13883, and *Salmonella enterica* ATCC 14028. The bacterial strains were cultured overnight at 37 °C in Mueller-Hinton Agar (MHA, Difco). The fungus was cultured at 25 °C in Sabouraud dextrose agar (SDA, Difco). 

#### 3.6.2. Disc diffusion method

The *in vitro* antimicrobial activity of the *T. riparia* essential oil was determined by the agar disk diffusion method. Briefly, 100 µL of suspension of the tested microorganism, containing 10^8^ colony-forming units (cfu)/mL of bacterial cells and 10^4^ cfu/mL spores of the fungus, respectively. Sterile filter-paper discs (Whatman No. 6.25 mm in diameter) were impregnated with 10 μL of the oil and placed on the inoculated plates. Discs without a sample were used as a negative control. Chloramphenicol, gentamicin, penicillin, and tetracycline discs were used as positive references for the bacteria and fungus, respectively to compare the sensitivity of the strains and isolates. The plates were incubated at 37 °C for 24 h for the bacterial strains, and at 37 °C for 48 h for the fungus. Antimicrobial activity was evaluated by measuring the diameter of the growth inhibition zones in millimeters (including the disc diameter of 6.25 mm) for the test organisms, and comparing to the controls. The inhibition zones were measured for three sample replicates, and the values presented are the means of three replicates [19]. 

#### 3.6.3. Microdilution MIC method

The minimal inhibitory concentrations of the oil for the strains were determined according to the M27-A2 and M7-A7 broth microdilution reference procedure of the NCCLS [20]. A stock solution of the essential oil was prepared in 2% Tween 80, and then serial dilutions were made from an initial concentration of 500 µg/mL. The 96-well plates were prepared by dispensing into each well, 95 µL of Sabouraud dextrose broth for yeasts, and Mueller-Hinton broth for bacteria; 100 µL of the oil (dissolved in 2% Tween); and 5 µL of the inoculum. The final volume in each well was 200 µL. [21]. Serial two-fold dilutions of the oil were done in a microdilution plate (96 wells) containing 100 µL of sterile medium. Next, the inoculum was added to each well. The microplates were incubated at 37 °C for 48 h for yeasts and 24 h for bacteria. Levofloxacin was used as a reference compound for antimicrobial activity. The MIC was defined as the lowest concentration that resulted in visible inhibition of growth. Minimal microbicidal concentrations were determined by subculturing 10 µL of the culture from each negative well and from the positive control, measured as described. [22].

### 3.7. Statistical analysis

Data from the 17 samples selected to be analyzed by chromatography, each one analyzed twice, were exported from the Agilent ChemStation as peak tables. The resulting peak tables were aligned using the software MSFACTs [23] using a retention time window of 0.08 min. After removing the peaks that appeared in fewer than five samples, since they were presumed to have a low discriminatory value, a matrix was obtained with 33 chromatographic profiles and 71 variables. This matrix was submitted to PCA. Sample 1 showed an anomalous behavior and was eliminated from the matrix, resulting in a final matrix with 31 chromatographic profiles and 71 variables. This matrix was submitted to PCA in order to assess the possibility of discrimination among samples collected in different seasons, and to PLS-DA to determine the most important peaks for the separation among the different seasons.

### 3.8. Principal components analysis (PCA)

PCA is a mathematical transformation of the original dataset into a smaller number of uncorrelated variables called principal components (PC). The coordinates of the samples relative to the PC axes are called scores, and loadings refer to the cosine of the angle between the original variables axis and the PC axis [24].

### 3.9. Partial least squares-discriminant analysis (PLS-DA)

PLS-DA is a classification method where a model is constructed between the matrix of the analytical results and a dummy matrix created to describe the class of samples. In this matrix, a sample belonging to each class is given the value of 1, and to all the other classes a value of -1 is assigned. In this study, the dummy matrix had 31 chromatographic profiles and 4 variables, one for each season. The model is obtained finding the components that maximize the covariance between the two matrices. With the model constructed and optimized, the regression coefficients were used to identify the most important variables to discriminate among the samples of different seasons. Large absolute values of the regression coefficient indicate which variables are more important in the model [25]. Both PCA and PLS-DA were carried out using the Unscrambler Version 9.1 software on the autoscaled matrix.

## 4. Conclusions

In general, the growing season affected the chemical composition and antimicrobial activities of the essential oil from *T. riparia* cultivated in southern Brazil. The analgesic activity of the oil was not affected by this seasonal variation. These differences can be attributed to the seasonal changes in rainfall. The oil from *T. riparia* showed good analgesic and antimicrobial activities. The production of essential oils and their utilization as potential natural sources for new phytomedicines could be of economic value. This species was acclimated in Brazil, and this is the first assessment of seasonal variations in the oil from its leaves.
